# The Buffer Effect of Different Wood Species and the Influence of Oak on Panel Composites Binders

**DOI:** 10.3390/polym12071540

**Published:** 2020-07-12

**Authors:** Franco Policardi, Marion Thebault

**Affiliations:** 1 Faculty of Electrical Engineering, University of Ljubljana, Tržaška cesta 25, 1000 Ljubljana, Slovenia; franc.policardi@fe.uni-lj.si; 2 Raziskovalni Inštitut za nove tehnologije in energetiko (R.I.N.T.E.) d.o.o., Stritarjeva 6/a, 4000 Kranj, Slovenia; 3 Kompetenzzentrum Holz (Wood K Plus), Altenberger Straße 69, 4040 Linz, Austria, c/o: Wood Carinthian Competence Center (W3C), Klagenfurter Straße 87-89, 9300 Sankt Veit an der Glan, Austria

**Keywords:** wood buffer capacity, oak wood, wood panel composites, melamine-urea-formaldehyde, urea-formaldehyde, wood adhesives

## Abstract

The buffer action of certain wood species can intensely affect the curing and hardening of some thermosetting wood adhesives. The present article presents a quantification of such buffering effects, determined under controlled conditions, in various wood species. The buffer capacity of oak has been found to be rather extreme and is likely to affect quite heavily the ability of urea-formaldehyde (UF) and melamine-urea-formaldehyde (MUF) wood panel adhesives in industrial operations. A variation of the buffer capacity of furnishes containing between 0% and 30% oak chips has been investigated. This was correlated with the internal bond (IB) strength of MUF bonded laboratory particleboards. The wood mixture buffering capacity increases with the oak content, while the panel IB strength decreases.

## 1. Introduction

Wood-based panels and composites are used as intermediates in a wide range of industrial applications, such as, for example, furniture, construction, packaging or do it yourself (DIY) products [1]. Particleboard, medium density fiberboard (MDF), oriented strand board (OSB) and plywood are the most common products in the wood panel industry, and are produced by means of synthetic adhesives (resins). Among the wide range of adhesives used, amino resins, a combination of formaldehyde and urea, as well as melamine, are the most important ones, which include the most popular urea-formaldehyde (UF) and melamine-urea-formaldehyde (MUF) resins [2]. As a structural component, the wood–adhesive bond is influenced by a variety of factors. Besides the physical and mechanical wood species properties, their chemical composition, e.g., wood extractives, can play a role in bonding wooden surfaces [3]. Some studies showed that interactions between the physico-chemical characteristics of resins and the properties of the wood substrate strongly affect bond quality [4,5]. Effective bonding and jointing techniques are therefore required to enhance the mechanical and physical performances of such materials. The formation and strength of glued joints are influenced by numerous wood characteristics. Most of wood species are acidic [6], and this characteristic affects their wettability [5], as well as their interaction with the resin [7].

In chemistry, the definition for a pH buffer is “an aqueous solution consisting of a mixture of a weak acid and its conjugate base, or vice versa”. In a wide variety of chemical applications, buffers are used as a mean of keeping pH at a nearly constant value, or to markedly minimize its variation. As a matter of fact, wood has a clear buffer effect and the latter strongly influences the hardening performance of water-carried wood adhesives that depend on pH variation [8,9].

Particleboard manufacturers often use wood from different sources, e.g., sawdust from sawmills, chipped small-diameter roundwood and recovered wood [1,10,11]. As the site of growth and trees’ vitality have a strong influence on the wood pH [12], these industrial resources are likely to change in pH and buffer capacities. For this reason, it is necessary to blend wood from different sources, and not to abruptly change the blend. To enhance the adhesion bond, one among other possible solutions is to pre-treat the wood chips and particles with either acid or base, or even with oxidant solutions such as peroxides. This enhances wood surface roughness and wettability [7], which further improves the wood-adhesive joint strength. However, this kind of pre-treatment also makes the process more complex and increases production costs.

The above used term “buffer capacity” refers to the wood’s resistance to a change in pH [6]. When a high buffer capacity wood is used, a high amount of acid (generally a H_2_SO_4_ solution) or alkali (generally a NaOH solution) must be added to change the overall mixture pH. It is important to mention that there is also a notable difference in pH and buffer capacity between the same tree wood and bark [13,14]. This observation enables one to realize that these resources should be separated and separately used in mixed panels manufacturing. More often than not, it is the buffer capacity fluctuation that causes most problems [15].

Wood species buffer capacity in wood panels production is a very difficult variable to control. The only chance to react to its changes and consequently adapt panels production and performance is through specific measurements. For instance, in order to obtain an optimum bond strength, the press time and temperature must be adjusted depending on the pH environment [6]. An interesting 2006 laboratory study demonstrated a strong relationship between the buffer capacity of particleboard furnish and its corresponding near-infrared (NIR) spectra via a multivariate analysis (chemometrics) [16]. In the same year, the buffer capacity of the wood resource was successfully predicted and applied using these techniques and models in an American particleboards plant.

As the rate of thermoset wood adhesives cross-linking is pH-dependent, these adhesives are sensitive, either to the substrate pH variation, or to the lack of it [17,18], and thus sensitive to the substrate buffer capacity [19]. Consequently, UF or MUF resins curing by a change in pH induction towards the acid field are badly affected by a wood species having a strong buffer capacity [20,21,22,23]. Park et al. [24] revealed that fiber acidity strongly affected the MDF panels internal bond (IB) strength with UF resin. Xing et al. [25] found a linear relationship between the gel time of UF resins and both absolute (acid buffering capacity-alkaline buffering capacity) and relative buffering capacity (acid buffering capacity/alkaline buffering capacity), as well as a linear relationship between the used species pH value and the UF resin gel time, which decreases as the pH value is decreasing. Industrial production tries to find a solution to this problem, most of the time using disproportionate amounts of acid-like hardening catalysts to succeed in hardening the resin, even vaguely, and sometimes not even properly. In this specific case, it is necessary to point out that short and long term damages can occur in the adhesive-wood joint when extreme pH values are reached [26,27,28]. Conversely, alkaline curing phenolic resins will be badly affected by most acidic woods, if, moreover, these have a strong buffer capacity. It has been demonstrated that no amount of alkali in the resin can bring it back to its optimal pH performance [28,29,30,31]. Thus, when wood buffering is a problem, it either retards or accelerate curing, so that the platen temperature in panels manufacturing process must be adapted to avoid pre-cure or over-cure.

Beside the complexity of these different assessments, the buffering capacity is highly variable among wood species [8]. Since 1973, wood species have been classified into two main groups: one having nearly no effect on the UF cure rate, and the other having some influence on the reaction [32].

The present paper starts with the buffering effect analysis of eight different wood species (three softwoods and five hardwoods) used in particleboard production, that may influence their gluing performances. Later, the higher or lower proportion of oak chips present in the wood composite furnish is here highlighted because of its particularly high buffer capacity. The work is specifically aimed at investigating the effect of oak chips addition on the buffering action exercised by the furnish, bonded with MUF adhesives. The gluing performances are evaluated by panels’ IB measurement.

## 2. Materials and Methods

Different freshly prepared wood species particles were placed in water in a proportion by weight 1:4 wood:water. The eight wood species were for softwoods: pine (*Pinus sylvestris*), spruce (*Picea abies*) and douglas fir (*Pseudotsuga menziesii*), and for hardwoods: hornbeam (*Carpinus betulus*), European beech (*Fagus sylvatica*), birch (*Betula alba*), eucalyptus (*Eucalyptus globulus*) and oak (*Quercus robur*). Demineralized water at pH 6 was used. The wood particles were ground to fine powders and fibers of a size ranging between 0.5 and 1 mm, and immersed in water according to the Sanderman and Rothkman method [33]. Then, 50.0 g of sawdust was mixed with 200 mL of water, and the mixture was brought to reflux for a brief period, then cooled down to 25 °C. The wood sawdust was then left in contact with the water for 24 h at a temperature of 23 °C. Afterwards, the solution was titrated by a 0.05 N solution of NaOH and/or H_2_SO_4_. The different wood species measured buffer capacity was then expressed as mEq (H^+^) and (OH^−^) to evolve the pH between 3 and 10 on 100 g of dry wood. In this case, and under these conditions, the buffer capacity is calculated as:

$$Buffer\;capacity=\frac{\left(v_{1}+v_{2}\right)\times 0.05\times 200}{250}$$

with

*v*_1_ = volume in ml of NaOH 0.05 N added up to reach a pH of 10.
*v*_2_ = volume in ml of H_2_SO_4_ 0.05 N added up to reach a pH of 3.

For each wood species and mixture, the pH of the solution was measured every 0.5 mL of NaOH and H_2_SO_4_ added solution. Each titration curve was repeated three times.

### 2.1. MUF Resin Preparation

A MUF resin with an (M + U)/F molar ratio of 1:1.2 and an M/U weight ratio of 47:53 (where M is melamine, U is urea and F is formaldehyde), was prepared as follows: to 71 parts of formurea (an industrial precondensate composed of 23% urea, 23% water and 54% formaldehyde) were added to 8 parts of urea and 15 parts of water. The pH was adjusted at 10.2 and the temperature increased up to 92–93 °C under continuous mechanical stirring, in reflux. As soon as the temperature was reached, the pH was lowered to 7.8 and the reaction continued at 92 °C for 25 min. A 22% NaOH water solution was then added to adjust the pH to 9.5. Then, 40 parts of melamine in 20 parts of water were added to the reaction mixture, followed by 2 parts of dimethylformamide. The temperature was maintained throughout, at 92 °C for 30 min. The pH slowly dropped to 7.2–7.5, and when the resin water tolerance reached a value of 180%, 21 parts of urea premixed with 5 parts water were added, while the pH was raised again to 9.5. The reaction continued until the water tolerance was lower than 150% (pH = 7.7), and after that the pH was adjusted to 10.0–10.2, by adding 22% NaOH water solution. The resin was finally cooled and stored. The resulting viscosity, measured with a Brookfield RV viscometer (Brookfield Ametek, Middleboro, MA, USA), was 220 mPa.s, with a solid content of 55.8%. The gel time at 100 °C with 2 wt% ammonium sulphate (NH_4_)_2_SO_4_ hardener was 95 s.

### 2.2. UF Resin Preparation

After this, 1500 g of formurea (UF concentrate) composed of 47 wt% formaldehyde, 21 wt% urea and 32 wt% water was used. Formurea was dissolved in 90 g of distilled H_2_O in a 3 L round bottom flask equipped with a condenser, mechanical stirrer and thermometer. The pH of the solution was adjusted to 8.2–8.5, with 30% caustic soda solution, and the temperature was raised to 65 °C. An amount of 356 g first urea was then charged into the solution—the temperature was increased to 88 ± 1 °C and maintained for 50 min to carry out the hydroxymethylation reaction. Subsequently, the pH of the reaction mixture was adjusted to 4.8–5.0 through addition of formic acid, and the condensation reaction was continued at 90 ± 1 °C to obtain the desired viscosity, namely above 200 mPa.s. At this point, a second amount of 588 g of urea was added to provide the final F/U molar ratio wanted of 1:1.2. The resin was finally cooled up to room temperature and the pH set to 8.2. The viscosity of the final mixture was 212 mPa.s, with a solid content of 62.3%. The gel time at 100 °C with 2 wt% ammonium sulphate (NH_4_)_2_SO_4_ hardener was 110 s.

### 2.3. Particleboard Testing

The glue mixes were prepared by adding 3% ammonium sulphate (NH_4_)_2_SO_4_ hardener on resin solids. Duplicate one-layer laboratory particleboard of 350 × 310 × 14 mm were prepared using industrial wood chips, composed of an oak and pine furnish in different proportions, as outlined in Table 1. The resin load used was 10% of total MUF solids content on dry wood. The resinated furnish mat was hot pressed for 5 min. The cycle consisted of a 28 kg/cm^2^ maximum pressure for 2 min from platen contact to high pressure (the 2 min included also the maintenance time at high pressure), followed by a descending pressing cycle of 1 min at 12–14 kg/cm^2^, and ending with 2 min at 5–7 kg/cm^2^, all at 190 °C–195 °C. The final resinated chips moisture content was 12%. After light surface sanding, the panels were tested for dry IB strength. The final value is an average of five repetitions.

### 2.4. Buffer Capacity of Defibered Wood Mixtures and Glued with UF and MUF Adhesives

Samples of wood-UF and wood-MUF glue mixes, prepared according to the method described above (10% of total adhesive solids content on dry wood), were dried using an oven at 103 °C and ground to fine powders of a size ranging between 0.5 and 1 mm. The powders were then immersed in demineralized water at pH 6 and prepared with the same method described above for wood sawdust.

For each mixture, the pH of the solution was measured every 0.5 mL of NaOH and H_2_SO_4_ added solution. Each titration curve was repeated three times.

## 3. Results and Discussion

**Figure 1:**
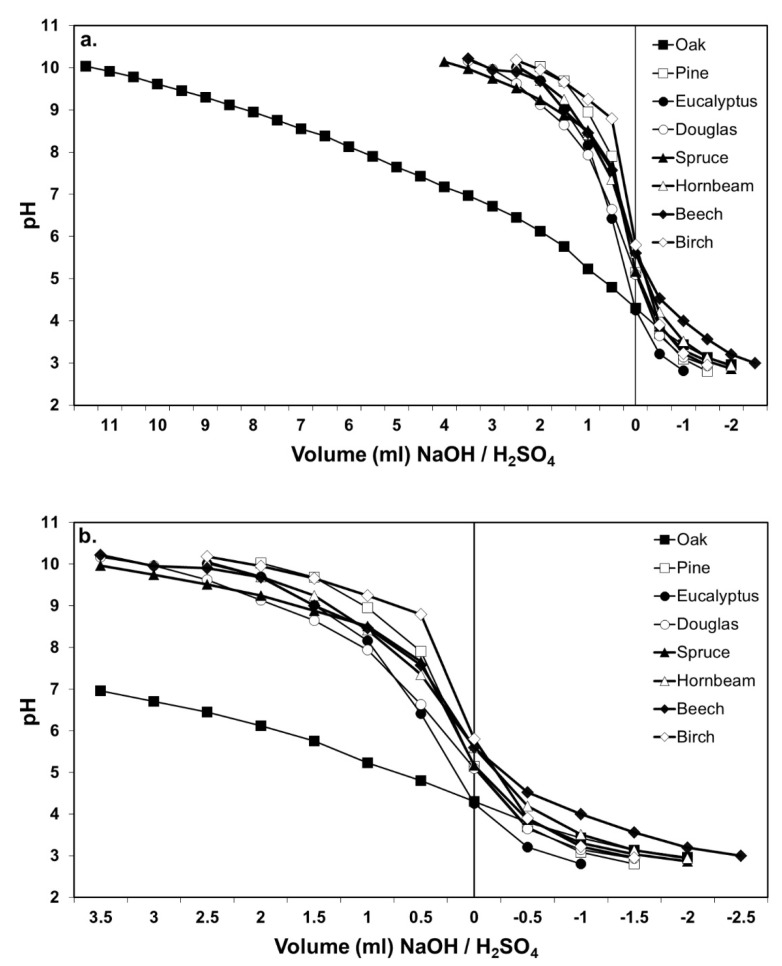
pH variation as a function of volume of 0.05N NaOH and/or 0.05 H_2_SO_4_ titration solution: (**a**) all wood species reported; (**b**) expanded detail of the lower part of the curves.

Figure 1a shows the buffer capacity graphs for the different investigated wood species. These results are similar to those previously exhibited in the study of Johns and Niazi [8]. This graph indicates the extreme buffer effect of oak chips in the alkaline domain, compared to the small differences between the buffer effects of the other wood species. Figure 1b has been drawn to better distinguish the buffer effect differences of the other examined wood species. The buffer capacity differences among the species other than oak are not great, and thus, a mix of chips and fibers of these species in different proportions will not markedly affect the performance of UF and MUF adhesives. Figure 1b describes how beech, spruce and douglas fir had a more marked buffer effect than the other species on the alkaline side; on the acid side, beech definitely distinguishes itself as the most buffering, and eucalyptus as the least. It is worth noting that, in the acid domain, the buffer capacity of oak is not particularly different from that of the other wood species, and it is even less than the one of beech.

**Figure 2:**
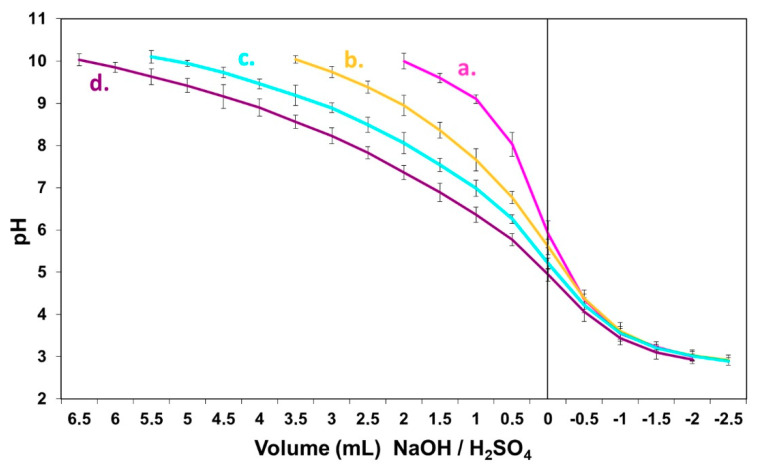
Buffer capacity effect of an oak/pine mixture furnish on the pH variation with: (**a**) 0% oak furnish-control; (**b**) 10% oak furnish; (**c**) 20% oak furnish; (**d**) 30% oak furnish.

As shown in Figure 1a, oak presents an extremely strong buffer capacity under alkaline conditions. The graph in Figure 2 highlights the specific oak furnish negative influence when mixed with other wood species furnish during UF and MUF bonded composite panels’ manufacturing process. Here, oak chips are mixed in a 10%, 20% and 30% proportion, respectively, to another low buffer capacity species (pine). It also shows how, as the proportion of oak increases, so does the buffer capacity of the total furnish, when compared to the buffer capacity of the control furnish, to which no oak material has been added.

**Figure 3:**
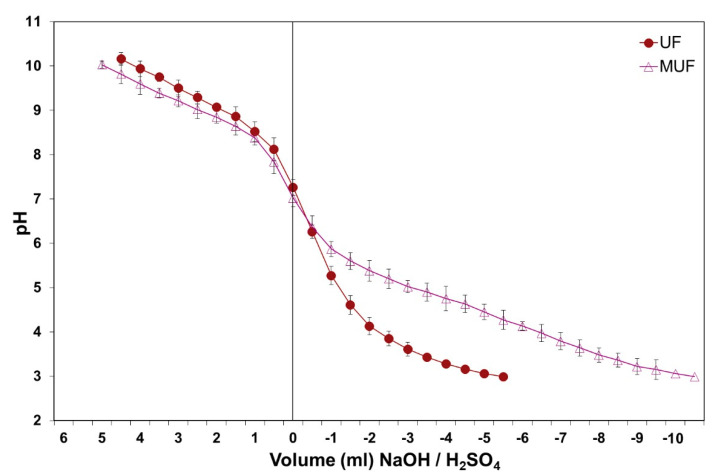
Adhesive type influence on the oak/pine mixture pH and buffer capacity, with 10% oak chips, after defibering and gluing.

Figure 3 presents the effects of 10% oak chips presence in the panel furnish buffer action on a MUF and an UF adhesive. For such resins, and in the presence of oak chips, the pH decreasing to hardening levels may cause the need to increase the acid quantity. This effect is very pronounced for the MUF resin (as melamine resins present a buffering action by themselves [21,34]). It becomes evident, then, that MUF resins will need extra acids for hardening in the presence of more than 10% oak in the wood furnish. An extreme effect has sometimes been observed in the industrial production process: the resin hardening is literally blocked, or so seriously slowed down, that the panel mechanical strength becomes faulty.

**Figure 4:**
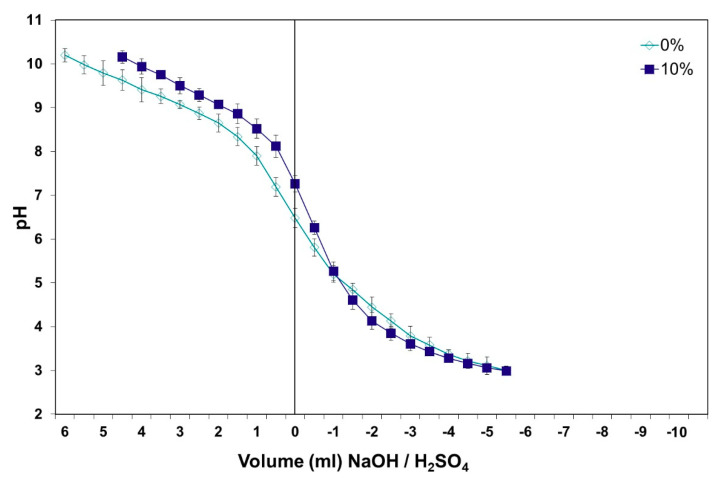
Oak species influence on the pH and buffer capacity of an oak/pine wood mixture, after defibering and gluing with a urea-formaldehyde (UF) resin.

The observed effect clearly appears in Figure 4, but is less damaging for UF resins; in the presence of 10% oak chips, the resin is less acidic and therefore less reactive—this is likely to also cause an adhesive hardening retardation. Meanwhile, at 10% of the oak chips, the problem appears to be easily curable for a UF resin; a higher percentage of oak chips will start to more seriously affect the UF resins hardening also. Such indications can be substantiated by laboratory particleboard testing, when the wood furnish comprises different proportions of oak chips.

UF and MUF adhesives generally harden by addition of an acid or acid-releasing salt, to reach a pH not lower than 4 [35]. Oak has a high buffer capacity maintaining the pH at medium levels, so curing a particleboard containing a certain proportion of oak is likely to become longer. Another study on UF demonstrated a linear relationship between the pH and the resin gel time [25]. The UF resin curing behavior is affected by the presence of wood raw materials in the system; this generally causes an enthalpy decrease [31]. It is then expected that the bonding quality of the particleboard is deteriorated by the presence of oak, due to an incomplete adhesive cure.

This effect can be observed from the dry IB strength of laboratory particleboard bonded with a standard MUF resin (Table 1).

The wood panel IB strength is the direct indicator of how good the bonding is. As the furnish percentage of oak chips increases, the IB strength value initially slowly decreases, then decreases progressively more and more, reaching very low values. This phenomenon attests to the negative effect of the oak buffer capacity; however, it can also be due to a difference of compressibility and surface area among pine and oak particles. Oak is denser than pine (in average 0.67 versus 0.55 with 12% moisture content [36]), and then harder to compress; some cavities between the chips can appear during the pressing stage, and weaken the gluing bond between them. Nevertheless, the whole density decreases with the oak content, with a maximal loss of only 8.5%. With an oak content of 10–20% in the furnish, the decrease in IB strength is relatively low, and the density remains almost the same (within standard deviation values).

## 4. Conclusions

The buffer capacities of eight different wood species (three softwoods and five hardwoods) have been evaluated in particleboard production. A notable difference has been observed in the case of oak, in which the buffer capacity under alkaline conditions is extremely strong. The buffer capacity effect of wood mixtures has been quantified in the case of oak wood chips used in wood panel composites such as particleboards. The presence of oak chips negatively influences UF and MUF resins bonding joints, and this has repercussions on the panels’ mechanical properties. While the use of up to between 10% and 20% of oak fibers and chips in the panel furnish just causes a moderate and still tolerable decrease in panel IB strength, the addition of higher percentages rapidly leads to a marked decrease in panel IB strength. This paper demonstrates that moderately acid-setting UF and MUF adhesives are notably and negatively influenced by such a strong oak wood furnish buffer capacity.

## Figures and Tables

**Table 1:** Internal bond (IB) strength of laboratory particleboard panels prepared using a melamine-urea-formaldehyde (MUF) resin on mixed wood furnish oak: pine at different ratios.

Wood Chips Oak:Pine	IB Strength (MPa)	Average Density Dry (kg/m^3^)
0:100	1.05 ± 0.06	0.710 ± 0.0094
10:90	0.99 ± 0.03	0.705 ± 0.0062
20:80	0.90 ± 0.05	0.700 ± 0.0102
30:70	0.72 ± 0.06	0.680 ± 0.0058
40:60	0.50 ± 0.11	0.670 ± 0.0113
50:50	0.34 ± 0.10	0.650 ± 0.0098
